# Integrated multiomic analyses: An approach to improve understanding of diabetic kidney disease

**DOI:** 10.1111/dme.15447

**Published:** 2024-10-26

**Authors:** Claire Hill, Amy Jayne McKnight, Laura J. Smyth

**Affiliations:** ^1^ Centre for Public Health, School of Medicine, Dentistry and Biomedical Science Queen's University Belfast Belfast UK

**Keywords:** diabetic kidney disease, epigenetics, genomics, lipidomics, metabolomics, proteomics, transcriptomics

## Abstract

**Aim:**

Diabetes is increasing in prevalence worldwide, with a 20% rise in prevalence predicted between 2021 and 2030, bringing an increased burden of complications, such as diabetic kidney disease (DKD). DKD is a leading cause of end‐stage kidney disease, with significant impacts on patients, families and healthcare providers. DKD often goes undetected until later stages, due to asymptomatic disease, non‐standard presentation or progression, and sub‐optimal screening tools and/or provision. Deeper insights are needed to improve DKD diagnosis, facilitating the identification of higher‐risk patients. Improved tools to stratify patients based on disease prognosis would facilitate the optimisation of resources and the individualisation of care. This review aimed to identify how multiomic approaches provide an opportunity to understand the complex underlying biology of DKD.

**Methods:**

This review explores how multiomic analyses of DKD are improving our understanding of DKD pathology, and aiding in the identification of novel biomarkers to detect disease earlier or predict trajectories.

**Results:**

Effective multiomic data integration allows novel interactions to be uncovered and empathises the need for harmonised studies and the incorporation of additional data types, such as co‐morbidity, environmental and demographic data to understand DKD complexity. This will facilitate a better understanding of kidney health inequalities, such as social‐, ethnicity‐ and sex‐related differences in DKD risk, onset and progression.

**Conclusion:**

Multiomics provides opportunities to uncover how lifetime exposures become molecularly embodied to impact kidney health. Such insights would advance DKD diagnosis and treatment, inform preventative strategies and reduce the global impact of this disease.

**Key points**

- Multiomic analyses have already provided, and continue to provide, insights into the molecular underpinnings of diabetic kidney disease.
- Work is needed to harness novel methods for improved data generation and advanced tools for data integratation to provide new insights into diabetic kidney disease.
- Integration of more diverse data types (such as co‐morbidity, environmental and demographic data) will facilitate a better understanding of kidney health inequalities.
- Global collaborations in the diabetic kidney disease field will facilitate larger, harmonised studies with increased diversity, to facilitate novel discoveries and ensure research is widely applicable.
- Efforts should be made to carefully detail sample collection, handling, storage, extraction, processing and bioinformatic pipelines to facilitate replication and comparison of results.

## 1 INTRODUCTION

Diabetes prevalence is growing, with the number of cases expected to rise from 537 million to 643 million between 2021 and 2030.^1^ Diabetes brings significant healthcare costs, estimated at £10.7 billion a year for the UK National Health Service, about 6% of the UK health budget,^2^ largely due to costs associated with treating complications. One such complication is diabetic kidney disease (DKD), developing in around 30%–50% of individuals living with diabetes; DKD is a leading cause of end‐stage kidney disease (ESKD).^3^, ^4^ DKD accounted for 30.7% of the 35.8 million disability‐adjusted life‐years (DALYs) caused by chronic kidney disease (CKD) in 2017, the largest proportion attributed to any cause.^5^

DKD often goes undetected until it reaches advanced stages.^6^ Kidney biopsies are the gold‐standard diagnosis method but are infrequently carried out due to their invasive nature.^7^ DKD is often diagnosed based on the co‐occurrence of diabetes and kidney disease.^8^, ^9^ DKD presentation is heterogeneous and does not always follow the classic CKD pathway.^9^, ^10^ For example, a large proportion of individuals with diabetes and reduced estimated glomerular filtration rate (eGFR) do not present with albuminuria.^11^, ^12^, ^13^, ^14^ Improved DKD diagnosis, particularly at an early stage, is required to reduce the personal and global burden by facilitating targeted prevention interventions and personalised therapeutics. However, major knowledge gaps, a lack of diagnostic tests and sub‐optimal screening, as well as regional variation in care, make this a challenge.^6^, ^15^, ^16^, ^17^, ^18^, ^19^, ^20^, ^21^ Moreover, inequalities in testing and disease management, with respect to age, sex, diabetes type and ethnicity, have been reported.^17^ Such inequalities can result in poor outcomes.^22^, ^23^, ^24^, ^25^ Multiomics provides an excellent opportunity to advance our molecular understanding of kidney disease, incorporating investigations into how environmental, social, economic and cultural exposures become molecularly embodied to impact kidney health. Multiomics plays an important role in overcoming kidney health inequalities.^26^, ^27^

**FIGURE 1:**
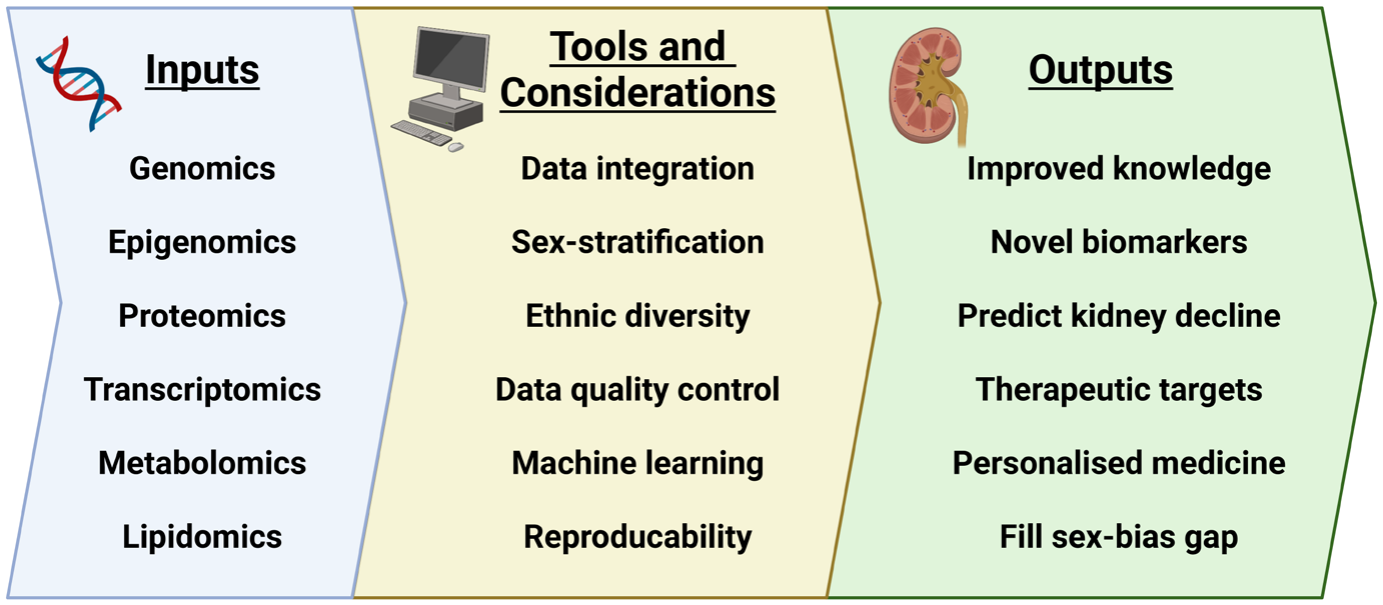
Multiomic workflow showing inputs, tools and considerations that lead to outputs to advance the diagnosis and treatment of diabetic kidney disease. Shown are common ‘omics’ but over 500 omics exist, such as foodomics, redoxomics, chronomics, pathogenomics and interactomics, each with their own potential to contribute towards our better understanding of DKD.

This review highlights advancements in the field of DKD multiomics, highlighting how innovation is improving our fundamental understanding of disease pathology, and revealing targets for potential therapeutic and/or biomarker development (Figure 1).

## 2 GENOMICS AND DKD

DKD heritability in T1D (Type 1 Diabetes) is estimated at 24%–42%, increasing to 59% when adjusting for sex, diabetes duration and age at diabetes diagnosis.^28^, ^29^ More stringent DKD definitions result in higher heritability estimates.^28^, ^29^ In T2D (Type 2 Diabetes), DKD heritability is ~8%–25%, reflecting more heterogeneity and a larger influence from external risk factors.^29^, ^30^, ^31^ Sandholm et al. (2023) summarised the status of genomic research in DKD.^29^ Forty‐one loci across 13 studies (Table S1) were significantly (*p* < 5 × 10^−8^) associated with DKD phenotypes,^29^, ^30^, ^31^, ^32^, ^33^, ^34^, ^35^, ^36^, ^37^, ^38^, ^39^, ^40^, ^41^ including ESKD, DKD and microalbuminuria, investigated in a range of T1D, T2D or combined populations. This paper summarised loci significantly associated with continuous traits estimated glomerular filtration rate (eGFR) (33 loci, 5 studies, Table S2),^30^, ^41^, ^42^, ^43^ and albuminuria (9 loci),^44^, ^45^, ^46^ in individuals with diabetes. These studies include studies across ethnicities,^37^ as well as in Japanese^38^ and African American^39^ cohorts.

Jin et al. identified three novel genetic variants (rs3128852, rs117744700 and rs28366355) significantly associated with DKD in multiple Korean cohorts.^47^ rs3128852 was an expression quantitative trait loci (eQTL) for *TRIM27* (Tripartite Motif Containing 27) (in whole blood) and *HLA‐A* (Human leukocyte antigens‐A) (in adipose‐subcutaneous tissues). rs28366355 was an eQTL for HLA‐group genes (in most tissues), with decreased expression of *TRIM27*, as well as a range of HLA genes, significantly associated with DKD.^47^ Table 1 outlines the biological plausibility for these genes to be involved in kidney function.

**TABLE 1:** Genes identified by Jin et al. via genomic analysis to be associated with DKD and literature highlighting the biological plausibility of their involvement in DKD.

Genes	Implication in kidney function	References
*TRIM27* (Tripartite Motif Containing 27)	*TRIM27* is involved in lupus nephritis (LN) and IgA nephropathy (IgAN), via two distinct pathways. DKD pathology differs from the immune‐related pathology of LN and IgAN, yet this work highlights that cellular pathways may overlap. Targeting *TRIM27* to mediate its expression may be useful to reduce injury of glomerular endothelial cells in LN, with the scope to explore this in DKD.	48, 49, 50, 51, 52
*HLA‐A* (Human leukocyte antigens‐A)	HLA has been associated with kidney function. Immune regulation is a key aspect of DKD.	53, 54

Whole genome (WGS) and whole exome (WES) sequencing provide new opportunities to explore rare and structural variants. Rare variants can influence DKD risk,^32^ likely contributing a larger effect compared to common variants.^55^ Harnessing WGS of siblings with T1D who were discordant for DKD (80 cases; 81 controls), variants present in protein‐coding and regulatory regions were associated with DKD; with replication in the Finnish Diabetic Nephropathy Study (FinnDiane) of unrelated T1D patients (1344 cases; 2187 controls).^56^ Variants were present within genes involved in podocyte function and within two protein kinase C gene isoforms, not previously associated with DKD,^56^ despite protein kinase C having been previously implicated in DKD.^57^

A 2024 preprint describes the WES and WGS analysis of 1097 individuals with T1D.^55^ Variants suggestively associated with T1D DKD (*THAP7* (THAP Domain Containing 7), *NAT16* (N‐Acetyltransferase 16), *LTA* (Lymphotoxin Alpha) and chromosome 18q12.3 (enhancer variant linked to *METTL4* (encoding Methyltransferase 4, N6‐Adenosine))) were replicated in FinnGen for CKD and/or DKD.^55^ Previous literature highlighting the plausibility of these genes implicated in DKD is shown in Table 2.^40^, ^55^, ^58^, ^59^, ^60^, ^61^, ^62^, ^63^

**TABLE 2:** Genes identified by Haukka et al. via WGS or WES to be associated with T1D‐DKD and literature highlighting the biological plausibility of their involvement in DKD.

Gene	Implication in kidney function
*THAP7* (THAP Domain Containing 7)	*THAP7* (rs369250) is an eQTL with *THAP7‐AS1* (THAP7 antisense RNA 1) in the Human Kidney Atlas (*p* = 8.488 × 10^−45^)^58^
*NAT16* (N‐Acetyltransferase 16)	*NAT16* has been shown to have significantly higher expression in idiopathic nodular glomerulosclerosis compared with healthy controls (Log_2_ fold change = 7.11, *p* = 1.67 × 10^−8^)^59^; a rare disease, where DKD kidney damage is seen in the absence of diabetes^60^
*LTA* (Lymphotoxin Alpha)	*LTA* (encoding tumor necrosis factor β (TNF‐β)) is an important factor in inflammation and apoptosis. An association between late diabetic complications and *LTA* (rs1041981) was suggested in 2008^61^ but has not been replicated. *LTA* is within HLA‐III,^62^ with Haukka et al. describing how the removal of the HLA regions in genome‐wide association studies (GWAS) may partly explain this lack of replication^55^
Chromosome 18q12.3 (enhancer variant linked to *METTL4* (encoding Methyltransferase 4, N6‐Adenosine))	The leading variant in 18q12.3 (rs16943099) was shown to disrupt the binding site of a podocyte‐specific transcription factor Mafb (MAF BZIP Transcription Factor B),^55^ with *mafb* recently discussed as a potential kidney disease treatment target.^63^ Moreover, a variant within *METTL4* (rs185299109) has been significantly associated with DKD (LINC00470/METTL4, *p* = 1.3 × 10^−8^)^40^

This work highlights several potential novel targets that may be manipulated to moderate kidney function and reduce damage in DKD. Moreover, next‐generation sequencing tools can increase the diagnostic yield for CKD, by accurately diagnosing cases with unknown etiology and in some instances updating primary diagnoses.^64^ Next‐generation and long‐read sequencing for DKD hold considerable promise to improve our understanding of DKD.

## 3 EPIGENETICS AND DKD

Epigenetic alterations, including DNA methylation, microRNAs and histone modifications, are known to cause changes to gene expression without altering the base DNA sequence. There is accumulating evidence to indicate that epigenetics has a role in both T1D and T2D development and progression.

DNA methylation, the most commonly studied epigenetic mechanism, can be described as a special chemical signature that provides important links between a person's lifestyle (diet, exercise, smoking, medication and environmental exposures) and their inherited risk factors for disease. DNA methylation occurs at cytosine bases, at cytosine‐phosphate‐guanine dinucleotide sites (CpGs) in the DNA sequence. These signatures are modifiable, often altering throughout the lifetime, and can be changed as a cause, or consequence of disease.^65^ There is also evidence to show that DNA methylation alterations remain in the blood for years following metabolic changes in the body,^66^ and so may be an underlying mechanism for metabolic memory.^67^, ^68^

Several investigations have been conducted into the links between DNA methylation and DKD using an epigenome‐wide association study approach (EWAS) which assess a portion of the ~28 million DNA methylation sites across the genome.^69^ The EWAS approach is cost‐efficient, high‐throughput and accurate with single‐base resolution.^70^ Illumina Infinium BeadChip technology is usually utilised via the Illumina 27 K array (~27,000 CpG sites), the Illumina 450 K array (~450,000 CpG sites), the Illumina EPIC array (~860,000 CpG sites) and the Illumina EPIC array v2.0 (~935,000 CpG sites).

The majority of EWAS for DKD have been carried out using blood‐derived DNA given the inaccessibility of kidney tissue,^7^, ^71^ but studies have been conducted using kidney tubule samples^72^ and DNA derived from saliva.^73^ To date, EWAS have identified several methylation loci associated with DKD, including the top‐ranked results from the primary analyses shown in Table 3.^72^, ^74^, ^75^, ^76^, ^77^, ^78^, ^79^, ^80^, ^81^, ^82^

**TABLE 3:** EWAS have identified several methylation loci associated with DKD, including the following top‐ranked results from the primary analyses. Full gene names are shown in the Abbreviations section.

CpG	Gene	*p*‐value (Ref)	Implication in kidney function
cg04861640	*ZNF187*	3.7 × 10^−24^ ^74^	Significant differential gene expression (*p* = 1.7 × 10^−5^) comparing tubulointerstitium to glomerular tissue in six transplant living donors^83^ and acute rejection following kidney transplantation (48 patients) (*p* = 2.8 × 10^−5^)^84^
			Gene expression changes were associated with eGFR in kidney biopsy and blood‐derived samples from people with IgA nephropathy,^85^ diabetes,^86^ and renal function in healthy individuals.^87^ Decreased renal function was associated with a decrease in *ZNF187* gene expression (22 racially diverse micro‐dissected human kidney samples with T2D DKD (*p* = 0.008)^86^
cg03546163	*FKBP5*	1.17 × 10^−24^ ^75^ and 3.6 × 10^−13^ ^76^	Gene expression was significantly reduced in DKD in all but one of the seven kidney datasets in Nephroseq.^76^ Higher *FKBP5* mRNA is associated with a pro‐inflammatory profile.^88^ Encodes FKBP51, regulator of Akt phosphorylation,^89^ nuclear factor‐kappa B (NF‐κB) activation^90^ and glucocorticoid receptor signalling.^91^ Hypomethylation at cg03546163 has previously been associated with DKD (*p* = 2.4 × 10^−9^)^77^ and kidney failure^75^ in T1D. Differential methylation of *FKBP5* is associated with multiple other diseases, including T2D and cardiometabolic risk^92^
cg26987613	*VPS52*	1.5 × 10^−1^ ^77^	
cg06207460	*AK3*	1.7 × 10^−25^ ^78^	
cg13525276	*TSHR*	3.87 × 10^−7^ ^79^	This is the thyroid‐stimulating hormone (TSH) receptor. An increase in TSH 12–24 months after kidney transplantation led to a significant decrease in eGFR^93^
cg20597486	*IFI16*	4.89 × 10^−6^ ^72^	An important transcriptional regulator which may modulate NF‐κB activation,^94^ is important in kidney function^95^
cg19693031	*TXNIP*	6.22 × 10^−14^ ^80^	Recently reviewed by Sun et al., and Choi and Park.^96^, ^97^ Exacerbates tubular fibrosis in the kidney.^98^ Redox protein is involved in mitochondrial reactive oxygen species (ROS) production, implicated in inflammasome activation and apoptosis in kidney injury.^99^ Increased level of *TXNIP* and nuclear translocation of TXN is associated with end‐stage renal disease.^100^ Regulates tubular autophagy and mitophagy in DKD through mTOR signalling^101^
cg16079347	*TMOD1*	2.87 × 10^−9^ ^81^	Involved in water regulation in the kidney.^102^ Identified in a protein–protein interaction network in DKD^103^
cg17944885	between *ZNF788P* and *ZNF625*‐*ZNF20*	padj = 0.0126 and *p* = 1.97 × 10^−44^ ^76^, ^82^	Differentially methylated in a range of EWAS, including multi‐ethnic studies.^104^, ^105^, ^106^ ZNF788P was significantly differentially expressed (*p* = 0.007) in CKD (renal biopsies)^107^

The largest EWAS conducted to date for T1D‐DKD was carried out as a meta‐analysis including 1304 carefully phenotyped individuals from the United Kingdom and Republic of Ireland (UK–ROI) and FinnDiane using the Infinium MethylationEPIC array; 32 differentially methylated CpG sites were identified, of which 21 were also shown to predict the development of kidney failure. In this analysis, three adjustment models were used including adjusting for age, sex, six white cell counts, then adding smoking status, before adding clinical covariates HbA1c, HDL cholesterol, triglycerides, diabetes duration and body mass index (BMI). Top‐ranked differentially methylated CpGs are shown in Table 4.^76^

**TABLE 4:** Top‐ranked differentially methylated CpGs identified in the largest EWAS to date.^76^

CpG	Gene	*p*‐value^76^	Implication in kidney function
cg25544931	Between *ZNF763* and *ZNF433‐AS1*	7.9 × 10^−19^	
cg17944885	Between *ZNF788P* and *ZNF625‐ZNF20*	8.91 × 10^−27^	As shown in Table 3
cg05710777	*LINC01800*	1.98 × 10^−11^	This CpG site overlapped with a binding site for the transcription factor AP‐2 alpha, previously implicated in DKD pathogenesis^76^, ^108^
cg21961721	*SLC27A3*	2.75 × 10^−10^	1.6‐fold higher expression in kidney glomeruli of individuals with DKD compared to healthy living donors (*p* = 6.61 × 10^−5^)^76^, ^109^
cg08150816	*NME7*	4.62 × 10^−10^	Significantly decreased expression in kidney glomeruli from individuals with DKD compared to healthy living donors (*p* < 0.01)^86^

A T2D‐DKD EWAS in 2021 explored DNA methylation profiles of 232 East Asian individuals,^81^ identifying associations cg16944159 within *COMMD1* (*p* = 8.2 × 10^−8^), cg16079347 within *TMOD1* (*p* = 2.87 × 10^−9^) and cg11530914 within *FHOD1* (*p* = 7.97 × 10^−8^).

Exciting opportunities also exist to utilise epigenetic data to generate biological ageing markers such as epigenetic clocks. Hishikawa et al. conducted a pilot study with 20 participants (8 non‐CKD (1 with diabetes) and 12 CKD (2 with diabetes)), determining that age acceleration measured by Hannum's clock^110^ negatively correlated with eGFR and eGFR slope (*p* < 0.05).^111^

Epigenetics provides an exciting focus, as these markers are modifiable. Indeed, it has been shown that drugs harnessed to slow kidney disease progression, such as Sodium‐glucose co‐transporter inhibitors (SGLT2i, namely Empagliflozin), may positively impact epigenetic status. Scisciola et al. (2023) highlighted that high glucose treatment of human ventricular cardiac myoblasts induced significant demethylation in the promoter regions of Nuclear Factor Kappa B (NF‐kB) and Superoxide Dismutase 2 (SOD2), mediated by an increase of Tet methylcytosine dioxygenase 2 (TET2) binding to the CpG island in these promoters.^112^ Empagliflozin prevents these demethylation changes by reducing TET2 binding and counteracts the altered gene expression. Pathways implicated (such as inflammation and redox) are important for kidney function, highlighting the potential for similar pathways to occur in the kidney with SGLT2i treatment.^113^, ^114^

## 4 TRANSCRIPTOMICS AND DKD

To determine the functional consequences of genetic or epigenetic variation, studies have harnessed transcriptomics, the study of RNA transcripts, via microarrays, quantitative‐PCR (qPCR) or RNA‐sequencing (RNA‐seq).^115^, ^116^ Investigations with respect to kidney disease, including DKD, have primarily explored messenger RNA (mRNA), non‐coding RNA (ncRNA) and micro‐RNA (miRNA).^116^, ^117^, ^118^, ^119^, ^120^, ^121^, ^122^ Contributions made by ncRNAs such as ribosomal RNA (rRNA), transfer RNA (tRNA), small nuclear RNA (snRNA), small nucleolar RNA (snoRNA) or long non‐coding RNA (lncRNA), have been the focus of recent reviews.^123^, ^124^, ^125^, ^126^, ^127^, ^128^ Yin et al. harnessed previously published DKD transcriptomic microarray datasets^121^, ^129^, ^130^ to reveal that *MMP2* (encoding Matrix Metalloproteinase 2) was significantly enriched in DKD. MMP2 contributes to fibrosis and inflammation in DKD and is potentially useful as an early urine marker.^131^ *miR‐106b‐5p* and *miR‐93‐5p* were identified as potential regulators of *MMP2* expression, highlighting potential use to prevent or delay DKD onset.^130^

Dwivedi et al. uncovered a potential novel biomarker of T1D‐DKD, via transcriptomic analysis of urinary extracellular vesicle (uEV) mRNA^132^ from macroalbuminuric (*N* = 17) and normoalbuminuric (*N* = 37) individuals. They identified 13 differentially expressed genes, with six associated with cellular stress. Extracellular vesicles (EVs) have been previously implicated in aspects of cellular stress.^133^, ^134^ The Dwivedi et al. stress score, based on the average normalized expression of six genes (Table 5), correlated with eGFR decline, even before albuminuria was observed (*R* ^2^ = 0.19, *p* = 2 × 10^−5^).^132^ Transcriptomic profiles largely did not vary between the sexes.^132^ The stress score has potential uses in non‐invasive early identification of individuals with declining kidney function and greater DKD risk.

**TABLE 5:** Six genes included in the Dwivedi et al. stress score in T1D‐DKD.

Gene	Encodes	Implication in kidney function
*GPX3*	Glutathione peroxidase 3	Produced in the kidney and deficiency linked to kidney disease^135^, ^136^
*NOX4*	NADPH oxidase 4	Nox‐dependent signalling pathways are implicated in kidney cell injury triggered by diabetic stimuli^137^
*MSRB1*	Methionine sulfoxide reductase B1	Redox control in mouse kidney^138^
*MSRA*	Methionine sulfoxide reductase A	Deficiency exacerbates kidney fibrosis^139^
*HRSP12* (*also known as UK114*)	Heat responsive protein 12	
*CRYAB*	Crystallin Alpha B	Marker gene for loop of henle. Small heat shock protein, which has been implicated in kidney disease^140^, ^141^

The development of single‐cell RNA sequencing (scRNA‐seq) allowed transcriptomes to be compared between individual cells,^142^ reviewed recently with a kidney focus.^143^, ^144^ Chen et al. have since used scRNA‐seq and spatial transcriptomics to create a DKD atlas.^145^ They utilised kidney tissue samples (DKD (*n* = 3), diabetes (n = 3) and non‐diabetic (n = 3)), identifying specific cell clusters, with significantly fewer endothelial cells and increased plasma cells associated with DKD. Genes were differentially expressed in DKD in several cell subsets. Additionally, crosstalk between cells differed more in DKD, identified via ligand‐receptor analysis, related to interactions between chemokines and fibroblasts, which authors suggested indicated that DKD renal fibrosis correlates with immune responses. Cell‐to‐cell interactions that were significantly different were confirmed via spatial transcriptomics.^145^

Single‐nucleus RNA sequencing (snRNA‐seq) is an alternative method that assesses single nuclei, utilised for the study of kidney disease and DKD.^146^, ^147^, ^148^, ^149^ Luo et al. utilised a previously published snRNA‐seq dataset,^147^ alongside microarray‐based transcriptomics, to identify four cell death pathways that were decreased in DKD.^150^ Their cell death‐related signature risk score (Table 6) distinguished between normal control and DKD individuals.^150^

**TABLE 6:** The core death genes used in the Luo et al. cell death‐related signature risk score.

Gene	Encodes	Implication in kidney function
*CASP1*	Caspase 1	Caspase‐1‐dependent inflammasome activation has a crucial function in promoting DKD^151^
*CYBB*	Cytochrome B‐245 Beta Chain	Differentially expressed in DKD tubuli. Involved in diabetic complications^86^
*PLA2G4A*	Phospholipase A2 Group IVA	Differentially expressed in DKD tubuli^86^
*CTSS*	Cathepsin S	Hub gene which contributes to the elucidation of DKD molecular pathogenesis^152^

## 5 LONG READ SEQUENCING: MAJOR TECHNICAL ADVANCE FOR GENOMICS, EPIGENOMICS AND TRANSCRIPTOMICS

Long‐read sequencing (voted Method of the Year 2023^153^), can read nucleotide sequences at a single molecule level, providing an unprecedented depth of information.^153^, ^154^, ^155^, ^156^ A 2023 study harnessed Nanopore sequencing to genotype RAGE (receptor for advanced glycation end products) polymorphisms, identifying 33 polymorphisms, including two novel low‐frequency mutations (positions 32181834 and 32181132 of chromosome 6) (healthy *N* = 71, T2D without nephropathy *N* = 86 and T2D with nephropathy *N* = 42).^157^ Four known mutations were validated by PCR‐RFLP (polymerase chain reaction‐restriction fragment length polymorphism), showing 99.75% concordance.^157^ Accumulation of RAGE results in oxidative stress and inflammation, as well as the upregulation of genes involved in fibrosis, scarring of glomeruli and epithelial–mesenchymal transition. This adds to the body of evidence supporting the involvement of RAGE in DKD pathology, potentially uncovering novel targets for intervention.^158^

Long‐read sequencing facilitated the Telomere‐to‐Telomere (T2T) human genome assembly, the first gapless assembly.^159^, ^160^ Long‐read sequencing was also utilised to generate the first draft of the Human Pangenome Reference; assemblies from 47 ethnically diverse individuals, with an aim of 350 genomes.^161^ Using this Pangenome, researchers reduced small variant discovery errors by 34% and increased the number of structural variants detected per haplotype by 104%. Moreover, they demonstrated the scope to improve RNA sequencing mapping.^161^ Tremendous scope exists to generate new DKD insights; utilising new datasets and reanalysing already generated data. Additionally, long‐read sequencing facilitates simultaneous genetic and epigenetic analysis, of DNA or RNA.^156^ It is possible to study molecular pathways at a higher resolution than ever before, potentially uncovering novel genes, pathways or modifications implicated in DKD pathology.

## 6 PROTEOMICS AND DKD

Proteomics is the quantification of proteins. Whilst genetics and epigenetics can define potential gene products, and transcriptomics reflects gene expression, proteomics provides functional insights.^162^ Proteomics can be analysed either via targeted approaches (such as antibody‐based multiplexing) or untargeted (quantifying as many proteins as possible, e.g., via mass spectrometry).^162^, ^163^ In 2011, Edwards et al. highlighted that 75% of protein research focused on the 10% of proteins known prior to the mapping of the human genome.^164^ In 2022, this annotation inequality remained, almost doubling since the human genome sequence was released.^165^, ^166^ A large portion of the proteome is largely neglected, driven by diversity in antibody‐based methods and quantitation.^162^ Mass spectrometry is a useful method, which can provide high specificity and isoform discrimination.^162^

Recent reviews have explored proteomics in DKD research.^116^, ^163^, ^167^, ^168^, ^169^, ^170^ Huang et al. utilised mass spectrometry to study proteomic profiles across patients with T2D (17 early DKD and 15 advanced DKD).^171^ All samples were formalin‐fixed paraffin‐embedded (FFPE) kidney specimens. NBR1 (Next to BRCA1 gene 1 protein) was significantly up‐regulated in early and advanced DKD, with VPS37A (Vacuolar protein sorting‐associated protein 37A) and ATG4B (Autophagy Related 4B Cysteine Peptidase) significantly down‐regulated. All three are closely related to autophagy, an intracellular stress response that plays a critical role in DKD progression.^172^ Serum levels of all three proteins and urine levels of NBR1 decreased with DKD progression,^171^ highlighting their potential as DKD biomarkers.

Unbiased proteomics was undertaken by Hirohama et al.,^173^ who utilised kidney samples from 23 individuals with DKD and ten healthy controls. A SomaScan assay was used to quantify the level of 1305 proteins, with levels of 14 proteins correlating with eGFR, and 152 proteins correlating with interstitial fibrosis. Matrix metalloprotease 7 (MMP7) showed the strongest association with both outcomes, with external validation. *MMP7* RNA levels also correlated with fibrosis, with external validation. scRNA‐seq revealed that proximal tubules, connecting tubules and principal cells were likely where increased tissue MMP7 expression was observed. Expression levels in blood samples revealed that plasma MMP7 levels correlated with kidney function and decline, revealing its potential use as a DKD biomarker.^173^ MMP7 has been highlighted as a sensitive biomarker for other kidney conditions (CKD and acute kidney injury), with future work needed to validate its use in urinary‐based DKD testing.^174^, ^175^

Zhao et al.^176^ assessed 75 individuals (30 DKD progressors; 45 DKD non‐progressors (up to 5‐year follow‐up)), harnessing liquid chromatography with tandem mass spectrometry (LC–MS/MS). Thirty‐eight proteins were significantly differentially expressed in DKD progressors relative to non‐progressors. PTGDS (Prostaglandin D2 Synthase), VWF (Von Willebrand factor), B2M (Beta 2 Microglobulin) and DAG1 (Dystroglycan 1) presented the most robust discriminatory power; however, combined models (including VWF, PTGDS, B2M, BT3A2 (Butyrophilin Subfamily 3 Member A2) and LCAT (lecithin cholesterol acyltransferase)) improved upon this discriminatory power. These authors highlighted previous studies implicating a number of these proteins, their encoding genes or paralogs, in kidney function, revealing the potential for them to be harnessed as novel biomarkers.^176^

Spatial proteomics is a useful method that utilises imaging to visualise proteins in their native cellular environment.^177^ Kondo et al. analysed 21 proteins in 23 tissue sections from 12 individuals. All participants had T2D (healthy kidneys *N* = 5, DKD stage IIA/IIB *N* = 4, DKD stage IIA‐B intermediate *N* = 2 and DKD class III *N* = 1).^178^ Using 21 proteins, 11 cell clusters were classified, relating to 10 known kidney compartments and cell types. DKD progression was associated with an increase in collagen IV deposition and the infiltration of inflammatory cells. This analysis advanced current understanding, determining that an increase in inflammatory cells and fibrosis was strictly co‐localised, suggesting co‐occurrence, rather than fibrosis occurring after inflammation.^178^ They also highlighted intra‐ and inter‐individual variability in molecular pathology of disease progression in kidney tissue, highlighting imitations of kidney biopsies in providing a whole‐kidney assessment of DKD severity,^178^ emphasising the need for advanced molecular biomarkers.

## 7 METABOLOMICS AND DKD

Metabolomics, an ever‐evolving technology that profiles metabolites in cells, tissues and biofluids, is routinely used for biomarker discovery. The main fluids used for metabolomic analysis are urine, serum and plasma.

Urine has several advantages in its lower protein and lipid content compared to alternative body fluids, but mainly in the ease of collection, which is non‐invasive.^179^ Urine metabolites were studied in individuals who took part in the CRIC study (*n* = 1001) reported by Kwan et al.^180^ Urinary metabolites were also examined by van der Kloet et al.^181^ and Liu et al.^182^ Van der Kloet et al.^181^ completed a 5.5‐year follow‐up study of 56 individuals with T1D. Liu et al. compared individuals with DKD in T2D and those with stable T2D.^182^ Serum analysis of individuals with DKD has been conducted by Zhang et al.,^183^ Chou et al.^184^ and Tofte et al.^185^ Serum metabolite analysis of 24 individuals with DKD in T2D was compared to those of 20 individuals with T2D and no DKD.^183^ Chou et al. compared the serum metabolites of 52 individuals with T2D‐DKD, with a 2‐year follow‐up.^184^ Tofte et al. investigated the serum metabolites of 637 individuals with T1D with a 5.5‐year follow‐up.^185^ Zhang et al. assessed the differences in plasma metabolites (132 healthy individuals; 132 individuals with T2D; 132 individuals with T2D‐DKD with 1‐year follow‐up).^186^ Tavares et al. investigated the plasma metabolites of 56 individuals with DKD over a 2.5‐year period.^187^ Niewczas et al.^188^ assessed the plasma metabolites of 40 individuals with T2D and 40 individuals with T2D who developed ESRD in the 8–12 years of follow‐up. Findings from these studies are summarised in Table 7.^189^, ^190^, ^191^

**TABLE 7:** Summary of key metabolites assessed in urine, serum and plasma and their association with kidney disease.

Metabolite	Source	Association with kidney function
3‐Hydroxyisobutyrate	Urine	Faster GFR decline, time to incident kidney failure and need for replacement therapy^180^
Acyl‐glycine and intermediates of tryptophan metabolism	Urine	Positively correlated with albuminuria and ultimately associated with T1D‐DKD progression^181^
Low levels of tyrosine	Urine	Higher T2D‐DKD risk^182^
Increased levels of trans‐4‐hydroxy‐l‐proline and 6‐aminocaproic acid	Serum	Early T2D‐DKD^183^
Tryptophan metabolism	Serum	Lowest levels associated with eGFR decline^184^
Branched‐chain amino acids, including isoleucine and valine	Serum	eGFR and lower risk of ESKD^185^
Tyrosine	Plasma	Decline of eGFR^186^
Decreased levels of norvaline	Plasma	Progression of DKD^187^
Decreased levels of aspartate	Plasma	Higher risk of mortality^187^
Increases in leucine and valine	Plasma	Lower risk of ESKD^188^
Higher levels of phenylacetyl‐glutamine and p‐cresol sulphate	Plasma	ESKD progression^188^
Higher levels of plasma/serum acylcarnitines	Plasma/serum	In individuals with DKD compared with individuals who had diabetes with no evidence of renal disease, which also predicted the progression of DKD^189^, ^190^, ^191^

Metabolomic results can be affected by genetics, environment and demographic factors, and often contradictory results can still arise from these studies. However, merging metabolomic data with additional omics data points can strengthen the research.

## 8 LIPIDOMICS AND DKD

Lipidomics is an emerging field of research focusing on the systematic and thorough study of lipids, a class of metabolites and their derivatives, in health and disease. It is often considered an extension of metabolomics, mirrored by the common use of high‐sensitivity mass spectrometry.^192^ Kidney disease and diabetes are known to result in great changes in the metabolism of both lipids and lipoproteins, which in turn contribute to disease progression as they play vital roles in the structure and function of cells, tissues and biofluids.^193^, ^194^, ^195^, ^196^

Prior to the extension of metabolomics to lipidomics, investigations of lipids in DKD have used traditional panels measuring cholesterol, triglycerides and lipoproteins. Advancements in both bioinformatic and mass spectrometry approaches have facilitated the enhanced levels of profiling now possible in plasma and at the tissue level. This enabled more detailed research, providing the ability to identify chemical characteristics, including class, chain length and degree of saturation.^196^ The ability to characterise lipids in this manner is valuable when assessing kidney disease, of which dyslipidaemia is a hallmark, as it may not be possible to capture lipid alterations that occur at a class level using the traditional approaches.^195^

Lipidomic investigations in DKD include an examination in a Pima American Indian cohort (*n* = 92) wherein it was discovered that one standard deviation increase in unsaturated free fatty acids was associated with a 0.54‐fold lower risk of DKD progression (*p* = 0.002), whilst taking glomerular filtration rate and albumin creatinine ratio into consideration.^197^ Additionally, an investigation in rat models of DKD compared to healthy models identified distinct lipidomic signatures in the DKD kidney, for example, in the significant increase of glyceride lipids in the kidney cortex and in the levels of lyso‐phospholipids and sphingolipids observed.^198^ The integration of lipidomic data with alternative omics, such as transcriptomes or proteomics, can enhance the datasets and aid in their interpretation, specifically of how changes reflect and impact the pathogenesis of kidney disease.^195^, ^199^ In murine models of DKD, 31 significantly different lipid metabolites were observed compared to the control group.^200^ The understanding of this dataset was enhanced through the addition of transcriptomic and network analyses. Lipidomics holds promise for novel DKD investigations as it has enhanced the scientific knowledge of metabolomic reprogramming in this disease.

## 9 MULTIOMIC DATA INTEGRATION

**FIGURE 2:**
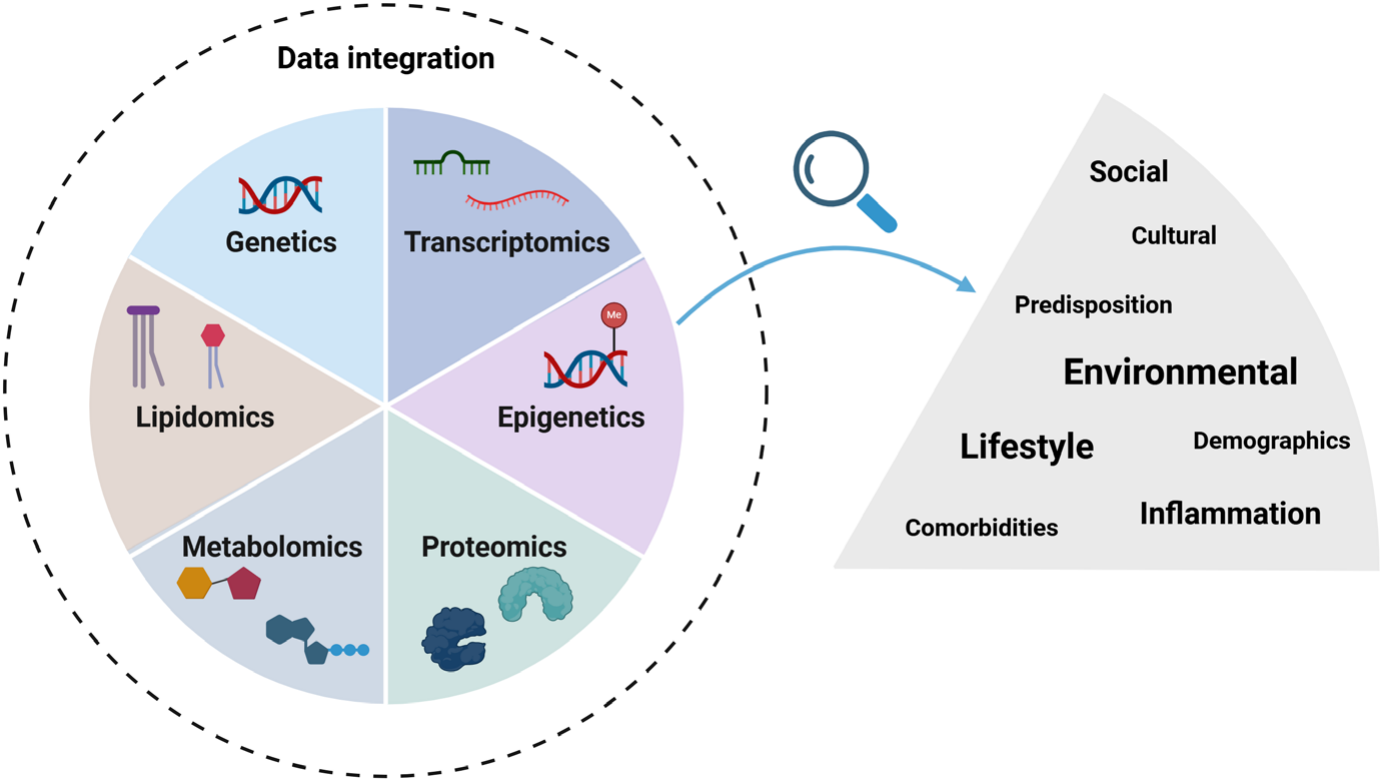
Data integration is looking at multiple ‐omics in a single analysis, allowing a more rounded picture of DKD pathology and its risk factors to be understood. Each ‐omic subtype is influenced by a specific combination of factors, such as demographics (age, sex and ethnicity), environmental exposures, lifestyle, genetic predisposition and co‐morbidities. Integrating multiple ‐omics allows complex patterns to be identified, potentially uncovering novel pathways disrupted during DKD or identifying factors (or combinations of factors) that increase an individual's risk of disease onset or progression.

As outlined in many of the examples above, studying ‐omic factors individually can provide meaningful insights; however, with comprehensive integration of datasets it is possible to detect complex interactions. This develops our understanding of how a network of ‐omic factors together disrupt biological pathways and influence disease. Each ‐omic will interrogate and account for specific factors, for example, genetic, lifestyle, environmental, inflammatory, co‐morbidity or demographic features, allowing complex interactions to be uncovered, advancing our fundamental understanding of DKD pathology and its risk factors (Figure 2). In 2022, our paper detailed studies that used integrated multiomic analysis to better our understanding of CKD, with a number of studies exploring DKD specifically.^116^

A review by Zhoi et al. explored the usefulness of artificial intelligence (AI) to assist integrated multiomic analysis in kidney disease, facilitating the development of personalised medicine.^201^ These authors highlight the need for valid data to obtain meaningful results. Considerations for biases, errors, missingness, lack of standardised reporting and differing methodologies must be made. They call for greater use of standardised references, protocols and quality control procedures to improve reproducibility. Collaborative resources are available to advance kidney disease research.^33^, ^35^, ^40^, ^75^, ^76^, ^159^, ^160^, ^161^, ^201^, ^202^, ^203^, ^204^, ^205^, ^206^, ^207^, ^208^, ^209^, ^210^, ^211^

A 2024 study by Li et al. demonstrated how integrated data analysis facilitated the discovery of a potential DKD biomarker. They utilised single‐cell RNA sequencing,^211^ proteomics,^212^ GWAS, metabolomics^213^ and database searches (such as GTex (www.gtexportal.org/), cis‐pQTL,^210^ NephroSeq (www.nephroseq.org/) and ProteomeXchange (www.proteomexchange.org)). They found that *AKR1A1* (aldo‐keto reductase family‐1 member A1) transcript and protein levels were significantly lower in the proximal tubule cells of DKD patients compared to controls. The metabolomic results suggested that insufficiency of AKR1A1 may lead to the accumulation of 3,4‐dihyroxybutyrate and glucoronate, key metabolites that predicted DKD in diabetic patients. Their work highlighted AKR1A1 and/or its metabolic network as potential DKD biomarkers.^214^

Jiang et al. described the metabolomic and peptidomic analysis of 766 individuals, across healthy controls, T2D patients, as well as T2D with early (microalbuminuria), overt (macroalbuminuria and typical diabetic glomerulopathy on renal biopsy) and grey‐zone (abnormal urinary microalbumin) DKD patients. Ten metabolites and six peptides were stepwise regulated at different DKD stages. Similar disrupted biological processes were highlighted in renal transcriptomics, as well as urine metabolomic and peptidomic data. Harnessing machine learning, they developed a stepwise prediction model, with 69.6%–89.9% of subjects within each group correctly classified in an external validation cohort.^215^

Integrated multiomic analysis in the context of DKD was also demonstrated by Kammer et al.^216^ This study utilised plasma proteomics, metabolomics and lipidomics, via the PROVALID study (PROspective cohort study in patients with type 2 diabetes mellitus for VALIDation of biomarkers). Patients with incident or early CKD were included. Comparisons were made between 258 individuals with a stable eGFR course and 223 individuals with rapid eGFR decline. KIM‐1 (kidney injury molecule 1) and NTproBNP (N‐terminal pro‐brain natriuretic peptide) were relevant predictors of eGFR trajectory. The authors note that baseline eGFR was an important clinical covariate and that lipidomics and metabolomics did not substantially improve discrimination models.^216^

Future work is needed to further expand this research to incorporate more diverse data types, such as environmental, multimorbidity and demographic data. This is important due to the influence environmental exposure, such as pollution, has on DKD risk.^217^, ^218^ DKD has also been identified as a key driver of multimorbidity,^219^, ^220^, ^221^ with complex interactions between conditions potentially impacting disease progression. Such information would advance our ability to provide personalised medicine or predict disease trajectory. Additionally, research has highlighted extensive inequalities with regard to kidney health and kidney disease care.^17^, ^27^ Kidney Research UK has declared that it is ‘Time to Act’ to overcome kidney‐health inequalities. Differences were described based on demographic factors such as age, sex, ethnicity, and deprivation.^222^ For example, in the United Kingdom, whilst women were more likely to be diagnosed with kidney disease, they were less likely to begin kidney replacement therapy.^27^ Additionally, adults whose ethnicity is Other, Black, Asian or Mixed had a higher likelihood of kidney failure and developed disease at a younger age compared to those of White ethnicity.^22^ Multiomic studies must incorporate analysis of diverse cohorts to ensure findings are widely applicable, and to improve equity in kidney care. Cohorts such as Our Future Health (https://ourfuturehealth.org.uk/) and All of Us (https://allofus.nih.gov/) are incorporating kidney measures and include multiomic data from individuals traditionally underrepresented in biomedical research.

## 10 AGEING AND DKD

As highlighted above, Phillips et al. reported that inequalities in disease management were observed with respect to age.^17^ Advancing age is a key risk factor for DKD.^25^, ^223^ Sex differences in biological features of ageing have the potential to impact kidney function decline.^23^, ^224^ For example, hormonal profiles change over the life course and may contribute towards DKD sex inequalities.^223^ Observations that female sex is protective against CKD progression may be limited to post‐menopausal age groups.^223^, ^225^ Recently, various markers of ageing have been investigated in the context of DKD. Farbe and Rangel explored traditional markers, such as eGFR, albuminuria and blood pressure, to predict DKD patients at risk of decline.^226^ Nagaraj et al. also utilised a range of clinical markers to develop a machine learning model to predict Kidney Age Index (KAI), with a 10‐year increase in mean KAI for DKD patients compared to healthy controls.^227^ Omic data have provided fresh opportunities to explore kidney disease in the context of ageing. For example, harnessing machine learning to analyse gene expression data, Luo et al. developed a cellular senescence‐related signature (SRS) utilising five hub genes (*LIMA1, ZFP36, FOS, IGFBP6, CKB*). A high SRS score was associated with lower eGFR and higher expression of fibrotic genes, as well as gene profiles suggesting suppression of mitochondrial function and higher immune cell infiltration.^228^

One marker of ageing that has garnered attention is telomere length. This topic was recently reviewed in the context of DKD by Hill et al., where a range of studies identifying associations between telomere length and kidney function, including CKD and DKD, were summarised.^209^ Telomere length has been shown to be significantly reduced in DKD versus control individuals, even after adjusting for age, duration of T1D, sex, BMI, and glycated haemoglobin (HbA1c) (*p* = 0.028).^229^ This paper incorporated multiomics, highlighting differential methylation in telomere‐related genes associated with DKD and ESKD, with these genes enriched for Wnt signalling. Wnt signalling has previously been implicated in both diabetes and DKD.^230^ Diabetes leads to the over‐activation of Wnt signalling, which promotes podocyte injury, podocyte epithelial‐mesenchymal transition and fibrosis.^231^ Harnessing previously published RNA‐sequencing datasets, genes where epigenetic dysregulation was correlated with altered gene expression identified potential diagnostic and therapeutic targets.^229^, ^231^ An exciting advancement was the measurement of telomere length at individual chromosome ends utilising long‐read sequencing,^232^ providing an opportunity to explore telomere length at unprecedented resolution. This is yet to be studied in the context of kidney disease.

Chromosomal aberrations beyond telomere shortening can impact kidney function; for example, mosaic loss of chromosomes. Specifically, mosaic loss of sex chromosomes may impact sex‐specific kidney function decline, which could contribute to DKD sex inequalities.^23^, ^223^, ^224^, ^225^ As individuals age, men experience mosaic loss of chromosome Y (mLOY) and women experience mosaic loss of chromosome x (mLOX) (preferentially in the inactivated X chromosome^233^). These are the most common somatic mutations acquired over the life course^234^, ^235^, ^236^; however, their causes and consequences are not well understood. Telomere shortening has been proposed as a contributing factor, as well as a lack of recombination or disrupted chromosomal segregation.^234^ Variants in telomere‐related genes (*TERT, MAD1L1*) have been significantly associated with mLOY.^237^ Interestingly, we have shown that *TERT* and *MAD1L1* are significantly differentially methylated (*p* ≤ 10^−8^) in DKD and ESKD (in T1D).^229^ mLOY and/or mLOX have been associated with diabetes, cardiovascular disease, CKD, renal complications in diabetes and transplant rejection.^236^, ^237^, ^238^, ^239^, ^240^, ^241^, ^242^ Moreover, mLOY was associated with variation near *TCL1A*,^243^ with *TCL1A* an effective marker of kidney transplantation tolerance.^244^ Scope exists to expand research in this field, especially with a DKD focus, to explore its impact on sex‐specific kidney function decline, potentially developing novel biomarkers or therapeutic targets.

## 11 CHALLENGES AND OPPORTUNITIES

**FIGURE 3:**
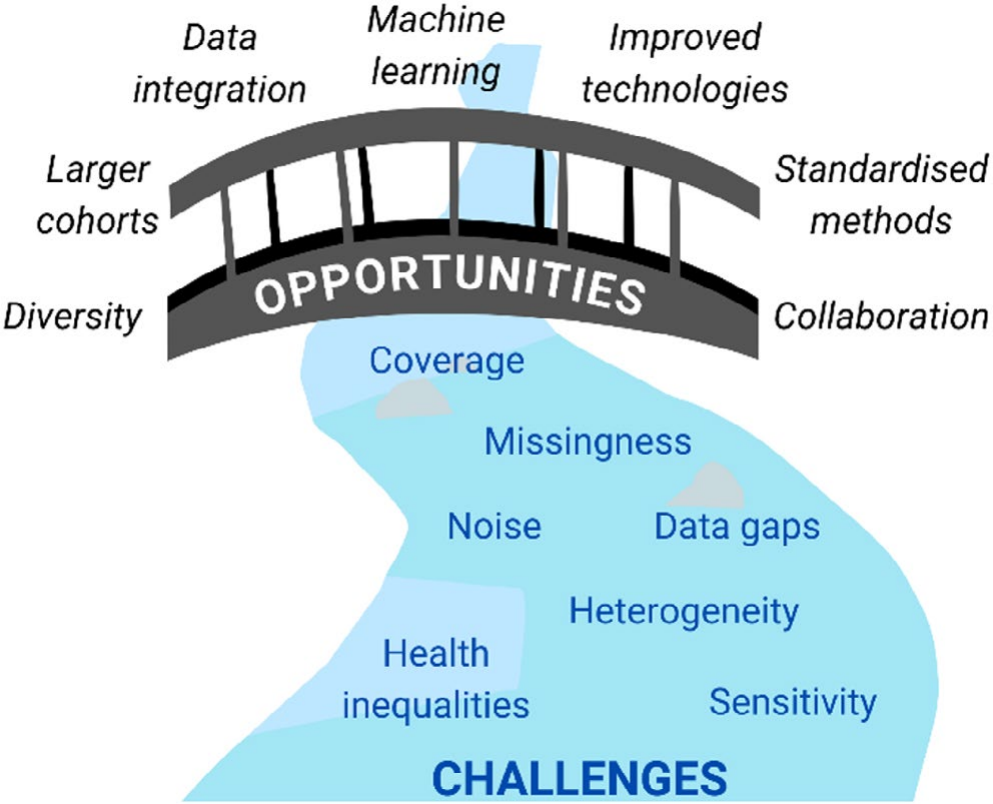
The DKD field is faced with challenges with respect to multiomic analysis. Data missingness and heterogeneity (a result of differing methods, tools, noise and sensitivity) can make integration of multiple datasets a challenge, exacerbated by data gaps with respect to ethnicity and sex. These data gaps contribute towards health inequalities and a poor understanding of diversity in DKD risk. Opportunities exist to overcome these challenges, harnessing novel methods, standardising techniques, utilising machine learning and collaborating globally to analyse larger cohorts with increased diversity.

Challenges remain in the field of multiomics (Figure 3), for example, the expensive nature of ‐omic analyses means studies may be limited to small sample sizes, which can hinder the power of analyses, resulting in small *p*‐values or effect sizes and limited results. Different ‐omics present different levels of resolution, and indeed, depending on budget, resolution can be increased or decreased, for example, read depth in transcriptomics or the size of array panels. Different sample types, such as blood, urine, tissue, cell culture or animal models can also bring complexity when integrating datasets, which acts as a source of heterogeneity. Additionally, ‐omic studies must utilise ethnically and socially diverse cohorts to ensure findings are widely applicable. Hill et al. summarised the challenges faced in the integration of multiomic datasets in CKD.^116^ It is recommended that, for optimum integration, ‐omic analyses should be conducted on samples that are collected at the same time point and are handled in a similar manner. Efforts should be made to carefully record details of sample collection, handling, storage, extraction, processing and bioinformatic analyses, as small changes can alter results. Global collaborations facilitate larger, harmonised studies with increased diversity, facilitating novel discoveries and widely applicable research.

## 12 CONCLUSIONS

With diabetes set to increase by almost 20% globally between 2021 and 2030, it is imperative that we uncover novel ways to manage complications, such as DKD. Improved tools are needed to identify those individuals living with diabetes who are at increased risk of DKD, so they can be closely monitored to prevent or slow DKD onset. Factors beyond genetics can influence DKD onset and progression. To help explain how changes in the environment affect disease traits, multiomic–environment interactions can be examined, possibly now due to the expanse in new technologies and data available.^245^, ^246^ Moreover, deeper insights into the underlying biology of DKD, as well as how it may present differently in men and women, and across different ethnicities, is required to ensure personalised care. Multiomics provides opportunities to generate knowledge and uncover novel patterns which could be utilised for novel biomarker discovery, therapeutic development or personalised medicine to help overcome DKD inequalities.

## AUTHOR CONTRIBUTIONS

CH—Conceptualization, investigation, visualization, writing—original draft. AJM—Conceptualization, writing—review and editing, resources, supervision. LJS—Conceptualization, investigation, writing—original draft, supervision. All authors agreed on the final version for submission.

## CONFLICT OF INTEREST STATEMENT

The authors declare no conflict of interest.

## Supporting information


Data S1.

Tables
