# Supplementary material for: Integrated multiomic analyses: An approach to improve understanding of diabetic kidney disease
Source: Diabet Med. 2024 Oct 26;42(2):e15447. doi: 10.1111/dme.15447 (PMC11733670; doi:10.1111/dme.15447)
Supplement: Supplementary file 1 — Data S1. [file DME-42-e15447-s001.docx]

**Supplementary tables**

**Supplementary Table 1:** Variants genome-wide significantly (p < 5 × 10^−8^) associated with DKD, summarised by Sandholm *et al.* (29).

| **Variant** |
| --- |
| rs7583877 |
| rs12437854 |
| rs4972593 |
| rs12523822 |
| rs56094641 |
| rs9942471 |
| rs72858591 |
| rs58627064 |
| rs142563193 |
| rs142671759 |
| rs4807299 |
| rs9622363 |
| rs75029938 |
| rs17577888 |
| rs55703767 |
| rs12615970 |
| rs142823282 |
| rs145681168 |
| rs118124843 |
| rs77273076 |
| rs551191707 |
| rs144434404 |
| rs115061173 |
| rs116216059 |
| rs191449639 |
| rs149641852 |
| rs183937294 |
| rs61983410 |
| rs113554206 |
| rs185299109 |
| rs72763500 |
| rs12917707 |
| rs538044833 |
| rs72831309 |
| rs141560952 |
| rs425827 |
| rs73038008 |
| rs77924615 |
| rs75733846 |
| rs559427701 |
| rs62202699 |

**Supplementary Table 2:** Variants genome-wide significantly (p < 5 × 10^−8^) associated with eGFR in diabetes, summarised by Sandholm *et al.* (29).

| **Variant** |
| --- |
| rs12917707 |
| rs11864909 |
| rs1974990 |
| rs10224002 |
| rs267738 |
| rs4665972 |
| rs10206899 |
| rs1047891 |
| rs4663171 |
| rs28817415 |
| rs10857147 |
| rs434215 |
| rs3812036 |
| rs34246779 |
| rs3101824 |
| rs11761603 |
| rs6464165 |
| rs9314272 |
| rs7033278 |
| rs80282103 |
| rs55917128 |
| rs963837 |
| rs2004649 |
| rs10899482 |
| rs2461700 |
| rs17631603 |
| rs11636251 |
| rs77924615 |
| rs9895661 |
| rs8096658 |
| rs6015028 |
| rs9607518 |
